# Supplementary material for: Association between obesity and urinary incontinence in older adults from multiple nationwide longitudinal cohorts
Source: Commun Med (Lond). 2023 Oct 11;3:142. doi: 10.1038/s43856-023-00367-w (PMC10567749; doi:10.1038/s43856-023-00367-w)
Supplement: Supplementary file 1 — Supplementary Data 1 [file 43856_2023_367_MOESM1_ESM.docx]

Supplementary Data 1. BMI, waist circumferences, and covariates of observations by sex in analyses

| Characteristics | HRS(N = 207,805) | | | | |  | ELSA(N = 98,158) | | | | |  | SHARE(N = 360,800) | | | | |  |
| --- | --- | --- | --- | --- | --- | --- | --- | --- | --- | --- | --- | --- | --- | --- | --- | --- | --- | --- |
|  | Female(N = 115,849) | | | Male(N = 91,956) | | | Female(N = 53,325) | | | Male(N = 44,833) | | | Female(N = 228,944) | | | Male(N = 183,915) | | |
|  | With UI (N = 18,070) | Without UI (N = 37,429) | p value | With UI (N = 6,065) | Without UI (N = 34,148) | p value | With UI (n = 5,629) | Without UI (N = 18,112) | p value | With UI (N = 1,973) | Without UI (N = 16,930) | p value | With UI (N = 5,488) | Without UI (N = 61,549) | p value | With UI (N = 2,203) | Without UI (N = 52,056) | p value |
| Age (years) | 70(60,79) | 64(57,74) | <0.001 | 74(65,81) | 64(57,74) | <0.001 | 69(62,77) | 66 (59,74) | <0.001 | 71(64,78) | 67 (60,74) | <0.001 | 73(63,81) | 63(57,72) | <0.001 | 75 (68, 81) | 64 (57, 72) | <0.001 |
| White (%) | 14,498 (80.2%) | 24,046(64.2%) | <0.001 | 4,594 (75.7%) | 23,865 (69.9%) | <0.001 | 5,469 (97.2%) | 17,479 (96.5%) | 0.018 | 1,891 (95.8%) | 16,334 (96.5%) | 0.120 | - | - | - | - | - | - |
| Educational attainments (%) |  |  |  |  |  |  |  |  | 0.210 |  |  | <0.001 |  |  | <0.001 |  |  | <0.001 |
| Less than secondary | 2,546(14.1%) | 5,681 (15.2%) | <0.001 | 901 (14.9%) | 4,377 (12.8%) | 0.003 | 1,644 (29.2%) | 5,137 (28.4%) |  | 524 (26.6%) | 3,439 (20.3%) |  | 3,522 (64.2%) | 30,391 (49.4%) |  | 1,231(55.9%) | 21,350 (41.0%) |  |
| Upper secondary and vocational training | 7,157 (39.6%) | 14,012(37.4%) |  | 3,175 (52.3%) | 17,486 (51.2%) |  | 2,551 (45.3%) | 8,392 (46.3%) |  | 866 (43.9%) | 8,464 (50.0%) |  | 1,345 (24.5%) | 20,475 (33.3%) |  | 605 (27.5%) | 19,074 (36.6%) |  |
| Tertiary | 3,463 (19.2%) | 6,610 (17.7%) |  | 1,201 (19.8%) | 6,885 (20.2%) |  | 876 (15.6%) | 2,702 (14.9%) |  | 474 (24.0%) | 3,933 (23.2%) |  | 621 (11.3%) | 10,683 (17.4%) |  | 367 (16.7%) | 11,632 (22.3%) |  |
| **Residence area (%)** |  |  |  |  |  |  |  |  | - |  |  | - |  |  | <0.001 |  |  | 0.43 |
| Rural | 5,163 (28.6%) | 8,878 (23.7%) | <0.001 | 1,596 (26.3%) | 25,183 (73.7%) | 0.013 | - | - |  | - | - |  | 1,348 (24.6%) | 17,057 (27.7%) |  | 613 (27.8%) | 15,195 (29.2%) |  |
| Urban | 12,679 (70.2%) | 28,063(75.0%) |  | 4,376 (72.2%) | 8,490 (24.9%) |  | - | - |  | - | - |  | 3,713 (67.7%) | 41,564 (67.5%) |  | 1,445(65.6%) | 34,469 (66.2%) |  |
| **Married and partnered (%)** | 9,916 (54.9%) | 19,191(51.3%) | <0.001 | 1,973 (32.5%) | 10,635 (31.1%) | 0.032 | 2,549 (45.3%) | 6,893 (38.1%) | <0.001 | 556 (28.2%) | 4,318 (25.5%) | 0.010 | 2,903 (52.9%) | 22,638 (36.8%) | <0.001 | 558 (25.3%) | 10,828 (20.8%) | <0.001 |
| **Number of Children (n)** | 2(2,4) | 2(2,3) | <0.001 | 2(2,4) | 2(1,3) | <0.001 | 2(1,3) | 2(1,3) | <0.001 | 2(1,3) | 2(1,3) | 0.076 | 2 (1, 3) | 2 (1, 3) | <0.001 | 2 (1, 3) | 2 (1, 3) | <0.001 |
| **Current smoking (%)** | 2,082 (11.5%) | 5,056 (13.5%) | <0.001 | 639 (10.5%) | 5,523 (16.2%) | <0.001 | 589 (10.5%) | 2,021 (11.2%) | 0.150 | 189 (9.6%) | 1,921 (11.3%) | 0.019 | 575 (10.5%) | 9,706 (15.8%) | <0.001 | 319 (14.5%) | 12,092 (23.2%) | <0.001 |
| **Ever smoked (%)** | 9,120 (50.5%) | 17,850(47.7%) | <0.001 | 4,045 (66.7%) | 21,843 (64.0%) | <0.001 | 3,370 (59.9%) | 10,003 (55.2%) | <0.001 | 1,388 (70.3%) | 11,602 (68.5%) | 0.110 | 1,579 (28.8%) | 20,852 (33.9%) | <0.001 | 1,330(60.4%) | 32,707 (62.8%) | 0.047 |
| **Alcohol consumption (%)** | 8,650 (47.9%) | 18,717(50.0%) | <0.001 | 3,311 (54.6%) | 22,158 (64.9%) | <0.001 | 4,176 (74.2%) | 14,189 (78.3%) | <0.001 | 1,564 (79.3%) | 14,371 (84.9%) | <0.001 | 2,770 (50.5%) | 34,493 (56.0%) | <0.001 | 1,363(61.9%) | 35,145 (67.5%) | <0.001 |
| **Physically active (%)** | 15,298 (84.7%) | 35,396(94.6%) | <0.001 | 5,120 (84.4%) | 32,939 (96.5%) | <0.001 | 5,169 (91.8%) | 17,690 (97.7%) | <0.001 | 1,804 (91.4%) | 16,622 (98.2%) | <0.001 | 4,747 (86.5%) | 60,324 (98.0%) | <0.001 | 1,842(83.6%) | 51,235 (98.4%) | <0.001 |
| **History of diseases** |  |  |  |  |  |  |  |  |  |  |  |  |  |  |  |  |  |  |
| Hypertension (%) | 11,848 (65.6%) | 21,893(58.5%) | <0.001 | 4,271 (70.4%) | 20,195 (59.1%) | <0.001 | 2,723 (48.4%) | 7,055 (39.0%) | <0.001 | 1,057 (53.6%) | 7,426 (43.9%) | <0.001 | 3,056 (55.7%) | 24,433 (39.7%) | <0.001 | 1,134(51.5%) | 19,393 (37.3%) | <0.001 |
| Diabetes (%) | 4,987 (27.6%) | 8,504 (22.7%) | <0.001 | 2,203 (36.3%) | 8,461 (24.8%) | <0.001 | 738 (13.1%) | 1,562 (8.6%) | <0.001 | 350 (17.7%) | 2,265 (13.4%) | <0.001 | 1,198 (21.8%) | 6,666 (10.8%) | <0.001 | 489 (22.2%) | 6,666 (12.8%) | <0.001 |
| Cancer (%) | 3,283 (18.2%) | 4,813 (12.9%) | <0.001 | 1,816 (29.9%) | 4,404 (12.9%) | <0.001 | 824 (14.6%) | 2,091 (11.5%) | <0.001 | 405 (20.5%) | 1,643 (9.7%) | <0.001 | 583 (10.6%) | 4,078 (6.6%) | <0.001 | 379 (17.2%) | 2,915 (5.6%) | <0.001 |
| Stroke (%) | 2,350 (13.0%) | 2,591 (6.9%) | <0.001 | 1,046 (17.2%) | 2,767 (8.1%) | <0.001 | 321 (5.7%) | 637 (3.5%) | <0.001 | 186 (9.4%) | 731 (4.3%) | <0.001 | 680 (12.4%) | 2,332 (3.8%) | <0.001 | 428 (19.4%) | 2,601 (5.0%) | <0.001 |
| **Cognitive impairment (%)** | 1,275 (7.1%) | 3,815 (10.2%) | <0.001 | 534 (8.8%) | 4,134 (12.1%) | <0.001 | 435 (7.7%) | 987 (5.4%) | <0.001 | 189 (9.6%) | 896 (5.3%) | <0.001 | 1,396 (25.4%) | 6,304 (10.2%) | <0.001 | 646 (29.3%) | 4,863 (9.3%) | <0.001 |
| **BMI (kg/m^2)** | 29.3±6.9 | 28.3±6.3 | <0.001 | 28.7±5.9 | 28.5±5.1 | 0.009 | 29.3±6.0 | 27.8±5.4 | <0.001 | 28.5±4.8 | 28.1±4.4 | 0.002 | 27.8±5.5 | 26.3±4.7 | <0.001 | 26.9±4.4 | 27.0±4.0 | 0.29 |
| **Waist circumferences (cm)** | 101.2±14.8 | 98.4±14.2 | <0.001 | 107.0±12.8 | 104.9±12.3 | <0.001 | 95.2±14.0 | 91.2±13.3 | <0.001 | 103.8±13.2 | 102.0±11.8 | <0.001 | - | - | - | - | - | - |
| Data are presented as mean ± SD or median (IQR), n (%) | | | | | | | | | | | | | | | | | | |
| * p value for differences between with UI and withou UI | | | | | | | | | | | | | | | | | | |
| HRS, the Health and Retirement Study; ELSA, the English Longitudinal Study of Ageing; SHARE, Survey of Health, Ageing and Retirement in Europe; BMI, body mass index; UI, urinary incontinence | | | | | | | | | | | | | | | | | | |
