# Supplementary material for: Association between obesity and urinary incontinence in older adults from multiple nationwide longitudinal cohorts
Source: Commun Med (Lond). 2023 Oct 11;3:142. doi: 10.1038/s43856-023-00367-w (PMC10567749; doi:10.1038/s43856-023-00367-w)
Supplement: Supplementary file 2 — Supplementary Data 2 [file 43856_2023_367_MOESM2_ESM.docx]

Supplementary Data 2. Association between BMI and waist circumferences, and prevalence of urinary incontinence

| Variables | Crude Model | | |  | Model 2 | | |  | Model 3 | | |  |
| --- | --- | --- | --- | --- | --- | --- | --- | --- | --- | --- | --- | --- |
|  | Female |  | Male |  | Female |  | Male |  | Female |  | Male |  |
|  | OR (95% CI) | p value | OR (95% CI) | p value | OR (95% CI) | p value | OR (95% CI) | p value | OR (95% CI) | p value | OR (95% CI) | p value |
| The HRS study | | | | | | | | | | | | |
| BMI (6 quantiles) | | | | | | | | | | | | |
| Quantile 1 | 0.807(0.708-0.920) | <0.001 | 1.849(1.535-2.227) | <0.001 | 0.622(0.532-0.727) | <0.001 | 1.630(1.323-2.009) | <0.001 | 0.589(0.498-0.697) | <0.001 | 1.298(1.023-1.647) | 0.032 |
| Quantile 2 | Reference | - | Reference | - | Reference | - | Reference | - | Reference | - | Reference | - |
| Quantile 3 | 0.894(0.787-1.016) | 0.085 | 0.902(0.766-1.063) | 0.220 | 1.015(0.873-1.179) | 0.848 | 1.125(0.939-1.348) | 0.202 | 1.049(0.894-1.230) | 0.557 | 1.125(0.921-1.374) | 0.249 |
| Quantile 4 | 1.066(0.928-1.224) | 0.364 | 0.866(0.725-1.036) | 0.115 | 1.389(1.179-1.638) | <0.001 | 1.157(0.950-1.410) | 0.147 | 1.424(1.196-1.696) | <0.001 | 1.057(0.849-1.315) | 0.622 |
| Quantile 5 | 1.237(1.067-1.434) | 0.005 | 0.940(0.777-1.137) | 0.522 | 1.909(1.598-2.280) | <0.001 | 1.410(1.141-1.742) | 0.001 | 1.936(1.603-2.337) | <0.001 | 1.268(1.002-1.603) | 0.048 |
| Quantile 6 | 1.687(1.442-1.973) | <0.001 | 1.234(0.974-1.488) | 0.086 | 3.129(2.575-3.801) | <0.001 | 2.192(1.729-2.780) | <0.001 | 3.070(2.496-3.776) | <0.001 | 1.903(1.463-2.475） | <0.001 |
| WC (6 quantiles) | | | | | | | | | | | | |
| Quantile 1 | 0.735(0.604-0.894) | 0.002 | 0.990(0.682-1.439) | 0.960 | 0.639(0.510-0.802) | <0.001 | 0.977(0.629-1.517) | 0.916 | 0.676(0.539-0.849) | 0.001 | 1.005(0.644-1.569) | 0.983 |
| Quantile 2 | Reference | - | Reference | - | Reference | - | Reference | - | Reference | - | Reference | - |
| Quantile 3 | 1.197(0.981-1.461) | 0.076 | 1.309(0.999-1.716) | 0.051 | 1.346(1.069-1.696) | 0.012 | 1.271(0.940-1.718) | 0.119 | 1.286(1.021-1.620) | 0.033 | 1.178(0.870-1.596) | 0.290 |
| Quantile 4 | 1.548(1.236-1.937) | <0.001 | 1.229(0.914-1.652) | 0.173 | 1.828(1.407-2.376) | <0.001 | 1.109(0.799-1.540) | 0.537 | 1.669(1.283-2.171) | <0.001 | 0.945(0.677-1.318) | 0.738 |
| Quantile 5 | 1.851(1.479-2.316) | <0.001 | 1.626(1.227-2.155) | 0.001 | 2.465(1.888-3.218) | <0.001 | 1.390(1.015-1.903) | 0.040 | 2.226(1.702-2.910) | <0.001 | 1.127(0.819-1.552) | 0.463 |
| Quantile 6 | 2.525(1.974-3.230) | <0.001 | 2.132(1.591-2.858) | <0.001 | 3.945(2.922-5.326) | <0.001 | 2.216(1.600-3.070) | <0.001 | 3.243(2.395-4.392) | <0.001 | 1.569(1.122-2.194) | 0.008 |
| The ELSA study | | | | | | | | | | | | |
| BMI (6 quantiles) | | | | | | | | | | | | |
| Quantile 1 | 1.014(0.803-1.282) | 0.904 | 1.295(0.920-1.821) | 0.138 | 1.013(0.787-1.305) | 0.920 | 1.302(0.913-1.858) | 0.145 | 0.978(0.681-1.404) | 0.903 | 1.119(0.617-1.433) | 0.632 |
| Quantile 2 | Reference | - | Reference | - | Reference | - | Reference | - | Reference | - | Reference | - |
| Quantile 3 | 1.403(1.114-1.768) | 0.004 | 1.132(0.855-1.499) | 0.387 | 1.504(1.171-1.933) | 0.001 | 1.154(0.861-1.546) | 0.338 | 1.153(0.794-1.673) | 0.455 | 1.059(0.647-1.327) | 0.776 |
| Quantile 4 | 1.667(1.300-2.137) | <0.001 | 1.307(0.967-1.767) | 0.082 | 1.774(1.353-2.327) | <0.001 | 1.381(1.009-1.892) | 0.044 | 1.850(1.255-2.726) | 0.002 | 1.184(0.660-1.445) | 0.422 |
| Quantile 5 | 2.058(1.598-2.649) | <0.001 | 1.144(0.832-1.573) | 0.406 | 2.268(1.722-2.987) | <0.001 | 1.281(0.918-1.787) | 0.146 | 2.693(1.817-3.997) | <0.001 | 1.132(0.673-1.547) | 0.574 |
| Quantile 6 | 2.846(2.201-3.680) | <0.001 | 1.609(1.139-2.275) | 0.007 | 3.459(2.611-4.583) | <0.001 | 1.788(1.238-2.583) | 0.002 | 4.081(2.744-6.071) | <0.001 | 1.562(0.919-2.304) | 0.055 |
| WC (6 quantiles) | | | | | | | | | | | | |
| Quantile 1 | 0.646(0.491-0.850) | 0.002 | 1.048(0.508-2.159) | 0.899 | 0.647(0.483-0.868) | 0.004 | 1.120(0.534-2.350) | 0.764 | 0.923(0.696-1.225) | 0.579 | 1.181(0.414-3.371) | 0.756 |
| Quantile 2 | Reference | - | Reference | - | Reference | - | Reference | - | Reference | - | Reference | - |
| Quantile 3 | 1.167(0.879-1.548) | 0.285 | 0.869(0.534-1.412) | 0.570 | 1.137(0.843-1.535) | 0.400 | 0.898(0.545-1.482) | 0.675 | 1.275(0.935-1.738) | 0.124 | 1.049(0.504-2.182) | 0.898 |
| Quantile 4 | 1.550(1.135-2.116) | 0.006 | 0.929(0.576-1.499) | 0.763 | 1.436(1.030-2.002) | 0.033 | 0.843(0.513-1.386) | 0.501 | 1.493(1.077-2.071) | 0.016 | 0.900(0.442-1.836) | 0.773 |
| Quantile 5 | 1.912(1.367-2.673) | <0.001 | 1.206(0.753-1.932) | 0.435 | 1.872(1.312-2.672) | 0.001 | 1.078(0.660-1.762) | 0.764 | 1.582(1.127-2.220) | 0.008 | 1.025(0.509-2.067) | 0.944 |
| Quantile 6 | 2.935(2.046-4.211) | <0.001 | 1.506(0.936-2.423) | 0.091 | 3.000(2.045-4.402) | <0.001 | 1.311(0.796-2.160) | 0.288 | 2.052(1.436-2.931) | <0.001 | 1.206(0.587-2.478) | 0.610 |
| The SHARE study | | | | | | | | | | | | |
| BMI (6 quantiles) | | | | | | | | | | | | |
| Quantile 1 | 0.921(0.787-1.078) | 0.306 | 1.659(1.332-2.0667) | <0.001 | 0.830(0.701-0.984) | 0.032 | 1.508(1.206-1.885) | <0.001 | 0.845(0.684-1.043) | 0.117 | 1.350(1.021-1.785) | 0.035 |
| Quantile 2 | Reference | - | Reference | - | Reference | - | Reference | - | Reference | - | Reference | - |
| Quantile 3 | 1.241(1.051-1.464) | 0.011 | 0.793(0.646-0.974) | 0.027 | 1.235(1.036-1.473) | 0.019 | 0.896(0.726-1.105) | 0.303 | 1.266(1.015-1.580) | 0.037 | 0.912(0.703-1.184) | 0.490 |
| Quantile 4 | 1.445(1.227-1.702) | <0.001 | 0.917(0.751-1.121) | 0.399 | 1.441(1.211-1.714) | <0.001 | 1.123(0.915-1.376) | 0.267 | 1.435(1.158-1.778) | 0.001 | 1.092(0.848-1.406) | 0495 |
| Quantile 5 | 1.848(1.569-2.177) | <0.001 | 0.935(0.760-1.150) | 0.525 | 1.922(1.615-2.287) | <0.001 | 1.126(0.953-1.458) | 0.309 | 1.770(1.428-2.193) | <0.001 | 1.070(0.825-1.388) | 0.610 |
| Quantile 6 | 3.008(2.570-3.520) | <0.001 | 1.130(0.915-1.396) | 0.255 | 3.314(2.798-3.925) | <0.001 | 1.594(1.287-1.975) | <0.001 | 2.788(2.251-3.453) | <0.001 | 1.513(1.165-1.965) | 0.002 |
| Model 2 was adjusted for age, race (except for the SHARE), educational attainments, residence area（except for the ELSA）, marital status, number of children; Model 3 was further adjusted for current smoking, ever smoked, alcohol consumption, physically activity, hypertension, diabetes, stroke, cancer, and cognitive impairments. | | | | | | | | | | | |  |
| HRS, the Health and Retirement Study; ELSA, the English Longitudinal Study of Ageing; SHARE, Survey of Health, Ageing and Retirement in Europe; BMI, body mass index; WC, waist circumference | | | | | | | | | | | |  |
