# Supplementary material for: Association between obesity and urinary incontinence in older adults from multiple nationwide longitudinal cohorts
Source: Commun Med (Lond). 2023 Oct 11;3:142. doi: 10.1038/s43856-023-00367-w (PMC10567749; doi:10.1038/s43856-023-00367-w)
Supplement: Supplementary file 3 — Supplementary Data 3 [file 43856_2023_367_MOESM3_ESM.docx]

**Supplementary Data 3.** Key STATA codes for the manuscript “Association between obesity and urinary incontinence in older adults from multiple nationwide longitudinal cohorts”

**Codes for Supplementary Data 2**

**Note 1: For HRS study**

*xtset hhidpn wave*

*xtlogit rurinai ib2.rbmi6 if ragender==1, or re*

*est store x1*

*xtlogit rurinai ib2.rbmi6 i.raracem i.agecat i.redu i.rrural i.rmarrital c.rchild if ragender==1, or re*

*est store x2*

*xtlogit rurinai ib2.rbmi6 i.raracem i.agecat i.redu i.rrural i.rmarrital c.rchild i.rtoilt i.rsmokev i.rsmoken i.rdrink i.rhibpe i.rdiabe i.rcancre i.rstroke i.rcogtot if ragender==1, or re*

*est store x3*

*xtlogit rurinai ib2.rmwaist6 if ragender==1, or re*

*est store x4*

*xtlogit rurinai ib2.rmwaist6 i.raracem i.agecat i.redu i.rrural i.rmarrital c.rchild if ragender==1, or re*

*est store x5*

*xtlogit rurinai ib2.rmwaist6 i.raracem i.agecat i.redu i.rrural i.rmarrital c.rchild i.rtoilt i.rsmokev i.rsmoken i.rdrink i.rhibpe i.rdiabe i.rcancre i.rstroke i.rcogtot if ragender==1, or re*

*est store x6*

*xtlogit rurinai ib2.rbmi6 if ragender==2, or re*

*est store x7*

*xtlogit rurinai ib2.rbmi6 i.raracem i.agecat i.redu i.rrural i.rmarrital c.rchild if ragender==2, or re*

*est store x8*

*xtlogit rurinai ib2.rbmi6 i.raracem i.agecat i.redu i.rrural i.rmarrital c.rchild i.rtoilt i.rsmokev i.rsmoken i.rdrink i.rhibpe i.rdiabe i.rcancre i.rstroke i.rcogtot if ragender==2, or re*

*est store x9*

*xtlogit rurinai ib2.rmwaist6 if ragender==2, or re*

*est store x10*

*xtlogit rurinai ib2.rmwaist6 i.raracem i.agecat i.redu i.rrural i.rmarrital c.rchild if ragender==2, or re*

*est store x11*

*xtlogit rurinai ib2.rmwaist6 i.raracem i.agecat i.redu i.rrural i.rmarrital c.rchild i.rtoilt i.rsmokev i.rsmoken i.rdrink i.rhibpe i.rdiabe i.rcancre i.rstroke i.rcogtot if ragender==2, or re*

*est store x12*

*outreg2 [x1 x2 x3 x4 x5 x6 x7 x8 x9 x10 x11 x12] using resultsHRSfinal.xls, excel replace eform*

**Note 2: For ELSA study**

*xtset idauniq wave*

*xtlogit rurinai ib2.rbmi6 if ragender==1, or re*

*est store x1*

*xtlogit rurinai ib2.rbmi6 i.raracem i.agecat i.redu i.rmarrital c.rchild if ragender==1, or re*

*est store x2*

*xtlogit rurinai ib2.rbmi6 i.raracem i.agecat i.redu i.rmarrital c.rchild i.rtoilt i.rsmokev i.rsmoken i.rdrink i.rhibpe i.rdiabe i.rcancre i.rstroke i.rcogtot if ragender==1, or re*

*est store x3*

*xtlogit rurinai ib2.rbmi6 if ragender==2, or re*

*est store x4*

*xtlogit rurinai ib2.rbmi6 i.raracem i.agecat i.redu i.rmarrital c.rchild if ragender==2, or re*

*est store x5*

*xtlogit rurinai ib2.rbmi6 i.raracem i.agecat i.redu i.rmarrital c.rchild i.rtoilt i.rsmokev i.rsmoken i.rdrink i.rhibpe i.rdiabe i.rcancre i.rstroke i.rcogtot if ragender==2, or re*

*est store x6*

*xtlogit rurinai ib2.rmwaist6 if ragender==1, or re*

*est store x7*

*xtlogit rurinai ib2.rmwaist6 i.raracem i.agecat i.redu i.rmarrital c.rchild if ragender==1, or re*

*est store x8*

*xtlogit rurinai ib2.rmwaist6 i.raracem i.agecat i.redu i.rmarrital c.rchild i.rtoilt i.rsmokev i.rsmoken i.rdrink i.rhibpe i.rdiabe i.rcancre i.rstroke i.rcogtot if ragender==1, or re*

*est store x9*

*xtlogit rurinai ib2.rmwaist6 if ragender==2, or re*

*est store x10*

*xtlogit rurinai ib2.rmwaist6 i.raracem i.agecat i.redu i.rmarrital c.rchild if ragender==2, or re*

*est store x11*

*xtlogit rurinai ib2.rmwaist6 i.raracem i.agecat i.redu i.rmarrital c.rchild i.rtoilt i.rsmokev i.rsmoken i.rdrink i.rhibpe i.rdiabe i.rcancre i.rstroke i.rcogtot if ragender==2, or re*

*est store x12*

*outreg2 [x1 x2 x3 x4 x5 x6 x7 x8 x9 x10 x11 x12] using resultsELSAfinal.xls, excel replace eform*

**Note 3: For SHARE study**

*xtset id wave*

*xtlogit rurinai ib2.rbmi6 if ragender==1, or re*

*est store x1*

*xtlogit rurinai ib2.rbmi6 i.agecat i.edunew i.hrural i.rmstatcat c.hchild if ragender==1, or re*

*est store x2*

*xtlogit rurinai ib2.rbmi6 i.agecat i.edunew i.hrural i.rmstatcat c.hchild c.rtoilt i.rsmokev i.rsmoken i.rdrink i.rhibpe i.rdiabe i.rcancre i.rstroke i.rcogtot if ragender==1, or re*

*est store x3*

*xtlogit rurinai ib2.rbmi6 if ragender==2, or re*

*est store x7*

*xtlogit rurinai ib2.rbmi6 i.agecat i.edunew i.hrural i.rmstatcat c.hchild if ragender==2, or re*

*est store x8*

*xtlogit rurinai ib2.rbmi6 i.agecat i.edunew i.hrural i.rmstatcat c.hchild c.rtoilt i.rsmokev i.rsmoken i.rdrink i.rhibpe i.rdiabe i.rcancre i.rstroke i.rcogtot if ragender==2, or re*

*est store x9*

*outreg2 [x1 x2 x3 x7 x8 x9] using resultsSHAREfinal.xls, excel replace eform*

**Codes for Figure 1**

**Note 1: For HRS study**

*mkspline rbmime=rbmi,cubic*

*xtset hhidpn wave*

*xtlogit rurinai rbmime* i.raracem i.agecat i.redu i.rrural i.rmarrital c.rchild c.rtoilt i.rsmokev i.rsmoken i.rdrink i.rhibpe i.rdiabe i.rcancre i.rstroke i.rcogtot if ragender==1*

*xblc rbmime1-rbmime4, covname(rbmi) at(15 15.1 15.2 15.3 15.4 15.5 15.6 15.7 15.8 15.9 16 16.1 16.2 16.3 16.4 16.5 16.6 16.7 16.8 16.9 17 17.1 17.2 17.3 17.4 17.5 17.6 17.7 17.8 17.9 18 18.1 18.2 18.3 18.4 18.5 18.6 18.7 18.8 18.9 19 19.1 19.2 19.3 19.4 19.5 19.6 19.7 19.8 19.9 20 20.1 20.2 20.3 20.4 20.5 20.6 20.7 20.8 20.9 21 21.1 21.2 21.3 21.4 21.5 21.6 21.7 21.8 21.9 22 22.1 22.2 22.3 22.4 22.5 22.6 22.7 22.8 22.9 23 23.1 23.2 23.3 23.4 23.5 23.6 23.7 23.8 23.9 24 24.1 24.2 24.3 24.4 24.5 24.6 24.7 24.8 24.9 25 25.1 25.2 25.3 25.4 25.5 25.6 25.7 25.8 25.9 26 26.1 26.2 26.3 26.4 26.5 26.6 26.7 26.8 26.9 27 27.1 27.2 27.3 27.4 27.5 27.6 27.7 27.8 27.9 28 28.1 28.2 28.3 28.4 28.5 28.6 28.7 28.8 28.9 29 29.1 29.2 29.3 29.4 29.5 29.6 29.7 29.8 29.9 30 30.1 30.2 30.3 30.4 30.5 30.6 30.7 30.8 30.9 31 31.1 31.2 31.3 31.4 31.5 31.6 31.7 31.8 31.9 32 32.1 32.2 32.3 32.4 32.5 32.6 32.7 32.8 32.9 33 33.1 33.2 33.3 33.4 33.5 33.6 33.7 33.8 33.9 34 34.1 34.2 34.3 34.4 34.5 34.6 34.7 34.8 34.9 35 35.1 35.2 35.3 35.4 35.5 35.6 35.7 35.8 35.9 36 36.1 36.2 36.3 36.4 36.5 36.6 36.7 36.8 36.9 37 37.1 37.2 37.3 37.4 37.5 37.6 37.7 37.8 37.9 38 38.1 38.2 38.3 38.4 38.5 38.6 38.7 38.8 38.9 39 39.1 39.2 39.3 39.4 39.5 39.6 39.7 39.8 39.9 40 40.1 40.2 40.3 40.4 40.5 40.6 40.7 40.8 40.9 41 41.1 41.2 41.3 41.4 41.5 41.6 41.7 41.8 41.9 42 42.1 42.2 42.3 42.4 42.5 42.6 42.7 42.8 42.9 43 43.1 43.2 43.3 43.4 43.5 43.6 43.7 43.8 43.9 44 44.1 44.2 44.3 44.4 44.5 44.6 44.7 44.8 44.9 45) reference(24) eform generate(pa1 or1 lb1 ub1)*

*twoway rarea lb1 ub1 pa1, bcolor(gs14) ///*

*|| line or1 pa1, clcolor(red) clwidth(medthick) ///*

*, ylabel(0(10)20, angle(0) nogrid) xlabel(15 (10) 45) ///*

*xtitle(BMI (kg/m^2)) ytitle(OR (95%CI)) title(Male) ///*

*name(male,replace) legend(order(2 "Expected OR" 1 "95% CI") rows(1))*

*mkspline rbmiop=rbmi,cubic*

*xtset hhidpn wave*

*xtlogit rurinai rbmiop* i.raracem i.agecat i.redu i.rrural i.rmarrital c.rchild c.rtoilt i.rsmokev i.rsmoken i.rdrink i.rhibpe i.rdiabe i.rcancre i.rstroke i.rcogtot if ragender==2*

*xblc rbmiop1-rbmiop4, covname(rbmi) at(15 15.1 15.2 15.3 15.4 15.5 15.6 15.7 15.8 15.9 16 16.1 16.2 16.3 16.4 16.5 16.6 16.7 16.8 16.9 17 17.1 17.2 17.3 17.4 17.5 17.6 17.7 17.8 17.9 18 18.1 18.2 18.3 18.4 18.5 18.6 18.7 18.8 18.9 19 19.1 19.2 19.3 19.4 19.5 19.6 19.7 19.8 19.9 20 20.1 20.2 20.3 20.4 20.5 20.6 20.7 20.8 20.9 21 21.1 21.2 21.3 21.4 21.5 21.6 21.7 21.8 21.9 22 22.1 22.2 22.3 22.4 22.5 22.6 22.7 22.8 22.9 23 23.1 23.2 23.3 23.4 23.5 23.6 23.7 23.8 23.9 24 24.1 24.2 24.3 24.4 24.5 24.6 24.7 24.8 24.9 25 25.1 25.2 25.3 25.4 25.5 25.6 25.7 25.8 25.9 26 26.1 26.2 26.3 26.4 26.5 26.6 26.7 26.8 26.9 27 27.1 27.2 27.3 27.4 27.5 27.6 27.7 27.8 27.9 28 28.1 28.2 28.3 28.4 28.5 28.6 28.7 28.8 28.9 29 29.1 29.2 29.3 29.4 29.5 29.6 29.7 29.8 29.9 30 30.1 30.2 30.3 30.4 30.5 30.6 30.7 30.8 30.9 31 31.1 31.2 31.3 31.4 31.5 31.6 31.7 31.8 31.9 32 32.1 32.2 32.3 32.4 32.5 32.6 32.7 32.8 32.9 33 33.1 33.2 33.3 33.4 33.5 33.6 33.7 33.8 33.9 34 34.1 34.2 34.3 34.4 34.5 34.6 34.7 34.8 34.9 35 35.1 35.2 35.3 35.4 35.5 35.6 35.7 35.8 35.9 36 36.1 36.2 36.3 36.4 36.5 36.6 36.7 36.8 36.9 37 37.1 37.2 37.3 37.4 37.5 37.6 37.7 37.8 37.9 38 38.1 38.2 38.3 38.4 38.5 38.6 38.7 38.8 38.9 39 39.1 39.2 39.3 39.4 39.5 39.6 39.7 39.8 39.9 40 40.1 40.2 40.3 40.4 40.5 40.6 40.7 40.8 40.9 41 41.1 41.2 41.3 41.4 41.5 41.6 41.7 41.8 41.9 42 42.1 42.2 42.3 42.4 42.5 42.6 42.7 42.8 42.9 43 43.1 43.2 43.3 43.4 43.5 43.6 43.7 43.8 43.9 44 44.1 44.2 44.3 44.4 44.5 44.6 44.7 44.8 44.9 45) reference(24) eform generate(pa or lb ub)*

*twoway rarea lb ub pa, bcolor(gs14) ///*

*|| line or pa, clcolor(red) clwidth(medthick) ///*

*, ylabel(0(10)20, angle(0) nogrid) xlabel(15 (10) 45) ///*

*xtitle(BMI (kg/m^2)) ytitle(OR (95%CI)) title(Female) ///*

*name(female,replace) legend(order(2 "Expected OR" 1 "95% CI") rows(1))*

*graph combine female male,title("Association between UI and BMI by Sex" "(in HRS study)")*

*graph export "hrs-rcs-bmi.png",replace*

**Note 2: For ELSA study**

*mkspline rbmime=rbmi,cubic*

*xtset idauniq wave*

*xtlogit rurinai rbmime* i.raracem i.agecat i.redu i.rmarrital c.rchild c.rtoilt i.rsmokev i.rsmoken i.rdrink i.rhibpe i.rdiabe i.rcancre i.rstroke i.rcogtot if ragender==1*

*xblc rbmime1-rbmime4, covname(rbmi) at(17 17.1 17.2 17.3 17.4 17.5 17.6 17.7 17.8 17.9 18 18.1 18.2 18.3 18.4 18.5 18.6 18.7 18.8 18.9 19 19.1 19.2 19.3 19.4 19.5 19.6 19.7 19.8 19.9 20 20.1 20.2 20.3 20.4 20.5 20.6 20.7 20.8 20.9 21 21.1 21.2 21.3 21.4 21.5 21.6 21.7 21.8 21.9 22 22.1 22.2 22.3 22.4 22.5 22.6 22.7 22.8 22.9 23 23.1 23.2 23.3 23.4 23.5 23.6 23.7 23.8 23.9 24 24.1 24.2 24.3 24.4 24.5 24.6 24.7 24.8 24.9 25 25.1 25.2 25.3 25.4 25.5 25.6 25.7 25.8 25.9 26 26.1 26.2 26.3 26.4 26.5 26.6 26.7 26.8 26.9 27 27.1 27.2 27.3 27.4 27.5 27.6 27.7 27.8 27.9 28 28.1 28.2 28.3 28.4 28.5 28.6 28.7 28.8 28.9 29 29.1 29.2 29.3 29.4 29.5 29.6 29.7 29.8 29.9 30 30.1 30.2 30.3 30.4 30.5 30.6 30.7 30.8 30.9 31 31.1 31.2 31.3 31.4 31.5 31.6 31.7 31.8 31.9 32 32.1 32.2 32.3 32.4 32.5 32.6 32.7 32.8 32.9 33 33.1 33.2 33.3 33.4 33.5 33.6 33.7 33.8 33.9 34 34.1 34.2 34.3 34.4 34.5 34.6 34.7 34.8 34.9 35 35.1 35.2 35.3 35.4 35.5 35.6 35.7 35.8 35.9 36 36.1 36.2 36.3 36.4 36.5 36.6 36.7 36.8 36.9 37 37.1 37.2 37.3 37.4 37.5 37.6 37.7 37.8 37.9 38 38.1 38.2 38.3 38.4 38.5 38.6 38.7 38.8 38.9 39 39.1 39.2 39.3 39.4 39.5 39.6 39.7 39.8 39.9 40 40.1 40.2 40.3 40.4 40.5 40.6 40.7 40.8 40.9 41 41.1 41.2 41.3 41.4 41.5 41.6 41.7 41.8 41.9 42 42.1 42.2 42.3 42.4 42.5 42.6 42.7 42.8 42.9 43 43.1 43.2 43.3 43.4 43.5 43.6 43.7 43.8 43.9 44 44.1 44.2 44.3 44.4 44.5 44.6 44.7 44.8 44.9 45) reference(25) eform generate(pa1 or1 lb1 ub1)*

*twoway rarea lb1 ub1 pa1, bcolor(gs14) ///*

*|| line or1 pa1, clcolor(red) clwidth(medthick) ///*

*, ylabel(0(10)20, angle(0) nogrid) xlabel(15 (10) 45) ///*

*xtitle(BMI (kg/m^2)) ytitle(OR (95%CI)) title(Male) ///*

*name(male,replace) legend(order(2 "Expected OR" 1 "95% CI") rows(1))*

*mkspline rbmiop=rbmi,cubic*

*xtset idauniq wave*

*xtlogit rurinai rbmiop* i.raracem i.agecat i.redu i.rmarrital c.rchild c.rtoilt i.rsmokev i.rsmoken i.rdrink i.rhibpe i.rdiabe i.rcancre i.rstroke i.rcogtot if ragender==2*

*xblc rbmiop1-rbmiop4, covname(rbmi) at(17 17.1 17.2 17.3 17.4 17.5 17.6 17.7 17.8 17.9 18 18.1 18.2 18.3 18.4 18.5 18.6 18.7 18.8 18.9 19 19.1 19.2 19.3 19.4 19.5 19.6 19.7 19.8 19.9 20 20.1 20.2 20.3 20.4 20.5 20.6 20.7 20.8 20.9 21 21.1 21.2 21.3 21.4 21.5 21.6 21.7 21.8 21.9 22 22.1 22.2 22.3 22.4 22.5 22.6 22.7 22.8 22.9 23 23.1 23.2 23.3 23.4 23.5 23.6 23.7 23.8 23.9 24 24.1 24.2 24.3 24.4 24.5 24.6 24.7 24.8 24.9 25 25.1 25.2 25.3 25.4 25.5 25.6 25.7 25.8 25.9 26 26.1 26.2 26.3 26.4 26.5 26.6 26.7 26.8 26.9 27 27.1 27.2 27.3 27.4 27.5 27.6 27.7 27.8 27.9 28 28.1 28.2 28.3 28.4 28.5 28.6 28.7 28.8 28.9 29 29.1 29.2 29.3 29.4 29.5 29.6 29.7 29.8 29.9 30 30.1 30.2 30.3 30.4 30.5 30.6 30.7 30.8 30.9 31 31.1 31.2 31.3 31.4 31.5 31.6 31.7 31.8 31.9 32 32.1 32.2 32.3 32.4 32.5 32.6 32.7 32.8 32.9 33 33.1 33.2 33.3 33.4 33.5 33.6 33.7 33.8 33.9 34 34.1 34.2 34.3 34.4 34.5 34.6 34.7 34.8 34.9 35 35.1 35.2 35.3 35.4 35.5 35.6 35.7 35.8 35.9 36 36.1 36.2 36.3 36.4 36.5 36.6 36.7 36.8 36.9 37 37.1 37.2 37.3 37.4 37.5 37.6 37.7 37.8 37.9 38 38.1 38.2 38.3 38.4 38.5 38.6 38.7 38.8 38.9 39 39.1 39.2 39.3 39.4 39.5 39.6 39.7 39.8 39.9 40 40.1 40.2 40.3 40.4 40.5 40.6 40.7 40.8 40.9 41 41.1 41.2 41.3 41.4 41.5 41.6 41.7 41.8 41.9 42 42.1 42.2 42.3 42.4 42.5 42.6 42.7 42.8 42.9 43 43.1 43.2 43.3 43.4 43.5 43.6 43.7 43.8 43.9 44 44.1 44.2 44.3 44.4 44.5 44.6 44.7 44.8 44.9 45) reference(25) eform generate(pa or lb ub)*

*twoway rarea lb ub pa, bcolor(gs14) ///*

*|| line or pa, clcolor(red) clwidth(medthick) ///*

*, ylabel(0(10)20, angle(0) nogrid) xlabel(15 (10) 45) ///*

*xtitle(BMI (kg/m^2)) ytitle(OR (95%CI)) title(Female) ///*

*name(female,replace) legend(order(2 "Expected OR" 1 "95% CI") rows(1))*

*graph combine female male,title("Association between UI and BMI by Sex" "(in ELSA study)")*

*graph export "elsa-rcs-bmi.png",replace*

**Note 3: For SHARE study**

*mkspline rbmime=rbmi,cubic*

*xtset id wave*

*xtlogit rurinai rbmime* i.agecat i.edunew i.rmstatcat i.hrural c.hchild c.rtoilta i.rsmokev i.rsmoken i.rdrink i.rhibpe i.rdiabe i.rcancre i.rstroke i.rcogtot if ragender==1*

*xblc rbmime1-rbmime4, covname(rbmi) at(15 15.1 15.2 15.3 15.4 15.5 15.6 15.7 15.8 15.9 16 16.1 16.2 16.3 16.4 16.5 16.6 16.7 16.8 16.9 17 17.1 17.2 17.3 17.4 17.5 17.6 17.7 17.8 17.9 18 18.1 18.2 18.3 18.4 18.5 18.6 18.7 18.8 18.9 19 19.1 19.2 19.3 19.4 19.5 19.6 19.7 19.8 19.9 20 20.1 20.2 20.3 20.4 20.5 20.6 20.7 20.8 20.9 21 21.1 21.2 21.3 21.4 21.5 21.6 21.7 21.8 21.9 22 22.1 22.2 22.3 22.4 22.5 22.6 22.7 22.8 22.9 23 23.1 23.2 23.3 23.4 23.5 23.6 23.7 23.8 23.9 24 24.1 24.2 24.3 24.4 24.5 24.6 24.7 24.8 24.9 25 25.1 25.2 25.3 25.4 25.5 25.6 25.7 25.8 25.9 26 26.1 26.2 26.3 26.4 26.5 26.6 26.7 26.8 26.9 27 27.1 27.2 27.3 27.4 27.5 27.6 27.7 27.8 27.9 28 28.1 28.2 28.3 28.4 28.5 28.6 28.7 28.8 28.9 29 29.1 29.2 29.3 29.4 29.5 29.6 29.7 29.8 29.9 30 30.1 30.2 30.3 30.4 30.5 30.6 30.7 30.8 30.9 31 31.1 31.2 31.3 31.4 31.5 31.6 31.7 31.8 31.9 32 32.1 32.2 32.3 32.4 32.5 32.6 32.7 32.8 32.9 33 33.1 33.2 33.3 33.4 33.5 33.6 33.7 33.8 33.9 34 34.1 34.2 34.3 34.4 34.5 34.6 34.7 34.8 34.9 35 35.1 35.2 35.3 35.4 35.5 35.6 35.7 35.8 35.9 36 36.1 36.2 36.3 36.4 36.5 36.6 36.7 36.8 36.9 37 37.1 37.2 37.3 37.4 37.5 37.6 37.7 37.8 37.9 38 38.1 38.2 38.3 38.4 38.5 38.6 38.7 38.8 38.9 39 39.1 39.2 39.3 39.4 39.5 39.6 39.7 39.8 39.9 40 40.1 40.2 40.3 40.4 40.5 40.6 40.7 40.8 40.9 41 41.1 41.2 41.3 41.4 41.5 41.6 41.7 41.8 41.9 42 42.1 42.2 42.3 42.4 42.5 42.6 42.7 42.8 42.9 43 43.1 43.2 43.3 43.4 43.5 43.6 43.7 43.8 43.9 44 44.1 44.2 44.3 44.4 44.5 44.6 44.7 44.8 44.9 45) reference(24.6) eform generate(pa1 or1 lb1 ub1)*

*twoway rarea lb1 ub1 pa1, bcolor(gs14) ///*

*|| line or1 pa1, clcolor(red) clwidth(medthick) ///*

*, ylabel(0(10)20, angle(0) nogrid) xlabel(15 (10) 45) ///*

*xtitle(BMI (kg/m^2)) ytitle(OR (95%CI)) title(Male) ///*

*name(male,replace) legend(order(2 "Expected OR" 1 "95% CI") rows(1))*

*mkspline rbmiop=rbmi,cubic*

*xtlogit rurinai rbmiop* i.agecat i.edunew i.rmstatcat i.hrural c.hchild c.rtoilta i.rsmokev i.rsmoken i.rdrink i.rhibpe i.rdiabe i.rcancre i.rstroke i.rcogtot if ragender==2*

*xblc rbmiop1-rbmiop4, covname(rbmi) at(15 15.1 15.2 15.3 15.4 15.5 15.6 15.7 15.8 15.9 16 16.1 16.2 16.3 16.4 16.5 16.6 16.7 16.8 16.9 17 17.1 17.2 17.3 17.4 17.5 17.6 17.7 17.8 17.9 18 18.1 18.2 18.3 18.4 18.5 18.6 18.7 18.8 18.9 19 19.1 19.2 19.3 19.4 19.5 19.6 19.7 19.8 19.9 20 20.1 20.2 20.3 20.4 20.5 20.6 20.7 20.8 20.9 21 21.1 21.2 21.3 21.4 21.5 21.6 21.7 21.8 21.9 22 22.1 22.2 22.3 22.4 22.5 22.6 22.7 22.8 22.9 23 23.1 23.2 23.3 23.4 23.5 23.6 23.7 23.8 23.9 24 24.1 24.2 24.3 24.4 24.5 24.6 24.7 24.8 24.9 25 25.1 25.2 25.3 25.4 25.5 25.6 25.7 25.8 25.9 26 26.1 26.2 26.3 26.4 26.5 26.6 26.7 26.8 26.9 27 27.1 27.2 27.3 27.4 27.5 27.6 27.7 27.8 27.9 28 28.1 28.2 28.3 28.4 28.5 28.6 28.7 28.8 28.9 29 29.1 29.2 29.3 29.4 29.5 29.6 29.7 29.8 29.9 30 30.1 30.2 30.3 30.4 30.5 30.6 30.7 30.8 30.9 31 31.1 31.2 31.3 31.4 31.5 31.6 31.7 31.8 31.9 32 32.1 32.2 32.3 32.4 32.5 32.6 32.7 32.8 32.9 33 33.1 33.2 33.3 33.4 33.5 33.6 33.7 33.8 33.9 34 34.1 34.2 34.3 34.4 34.5 34.6 34.7 34.8 34.9 35 35.1 35.2 35.3 35.4 35.5 35.6 35.7 35.8 35.9 36 36.1 36.2 36.3 36.4 36.5 36.6 36.7 36.8 36.9 37 37.1 37.2 37.3 37.4 37.5 37.6 37.7 37.8 37.9 38 38.1 38.2 38.3 38.4 38.5 38.6 38.7 38.8 38.9 39 39.1 39.2 39.3 39.4 39.5 39.6 39.7 39.8 39.9 40 40.1 40.2 40.3 40.4 40.5 40.6 40.7 40.8 40.9 41 41.1 41.2 41.3 41.4 41.5 41.6 41.7 41.8 41.9 42 42.1 42.2 42.3 42.4 42.5 42.6 42.7 42.8 42.9 43 43.1 43.2 43.3 43.4 43.5 43.6 43.7 43.8 43.9 44 44.1 44.2 44.3 44.4 44.5 44.6 44.7 44.8 44.9 45) reference(24.6) eform generate(pa or lb ub)*

*twoway rarea lb ub pa, bcolor(gs14) ///*

*|| line or pa, clcolor(red) clwidth(medthick) ///*

*, ylabel(0(10)20, angle(0) nogrid) xlabel(15 (10) 45) ///*

*xtitle(BMI (kg/m^2)) ytitle(OR (95%CI)) title(Female) ///*

*name(female,replace) legend(order(2 "Expected OR" 1 "95% CI") rows(1))*

*graph combine female male,title("Association between UI and BMI by Sex" "(in SHARE study)")*

*graph export "share-rcs-bmi.png",replace*

**Codes for Figure 2**

*mkspline rmwaistme=rmwaist,cubic*

*xtset hhidpn wave*

*xtlogit rurinai rmwaistme* i.raracem i.agecat i.redu i.rrural i.rmarrital c.rchild c.rtoilt i.rsmokev i.rsmoken i.rdrink i.rhibpe i.rdiabe i.rcancre i.rstroke i.rcogtot if ragender==1*

*xblc rmwaistme1-rmwaistme4, covname(rmwaist) at(68.58 69.215 69.342 69.85 70.485 71.12 71.755 72.39 73.025 73.66 74.295 74.676 74.93 75.565 75.6412 76.2 76.835 77.47 78.105 78.232 78.74 79.248 79.375 80.01 80.645 81.28 81.788 81.915 82.55 82.804 83.185 83.82 84.455 85.09 85.725 86.36 86.741 86.995 87.63 87.884 88.265 88.9 88.9508 89.408 89.535 89.789 90.17 90.805 91.44 91.567 91.7702 91.948 92.075 92.71 92.964 93.218 93.345 93.472 93.98 94.0054 94.0308 94.107 94.234 94.615 95.25 95.885 96.52 96.647 97.155 97.2312 97.3836 97.79 97.917 98.425 99.06 99.187 99.441 99.568 99.695 100.33 100.965 101.6 101.727 101.854 102.108 102.235 102.87 102.997 103.378 103.505 104.14 104.267 104.775 105.029 105.156 105.41 106.045 106.426 106.68 106.807 106.934 107.188 107.315 107.696 108.585 109.22 109.347 109.474 109.855 110.49 110.998 111.125 111.252 111.76 111.887 112.395 113.03 113.665 114.3 114.554 114.935 115.062 115.57 116.205 116.84 117.348 117.475 117.602 118.11 118.745 119.38 119.507 119.634 119.888 120.015 120.65 121.285 121.92 122.301 122.555 123.19 123.825 124.46 124.587 124.714 125.095 125.73 126.365 127 127.127 127.635 128.016 128.27 128.905 129.54 129.794 130.175 130.81 131.445 132.08 132.715 133.35 133.985 134.62 135.255 135.89 136.525 137.16 137.541 137.795 138.43 139.065 139.7) reference(95.25) eform generate(pa1 or1 lb1 ub1)*

*twoway rarea lb1 ub1 pa1, bcolor(gs14) ///*

*|| line or1 pa1, clcolor(red) clwidth(medthick) ///*

*, ylabel(0(5)10, angle(0) nogrid) xlabel(70 (10) 140) ///*

*xtitle(Waist Circumference (cm)) ytitle(OR (95%CI)) title(Male) ///*

*name(male,replace) legend(order(2 "Expected OR" 1 "95% CI") rows(1))*

*mkspline rmwaistop=rmwaist,cubic*

*xtset hhidpn wave*

*xtlogit rurinai rmwaistop* i.raracem i.agecat i.redu i.rrural i.rmarrital c.rchild c.rtoilt i.rsmokev i.rsmoken i.rdrink i.rhibpe i.rdiabe i.rcancre i.rstroke i.rcogtot if ragender==2*

*xblc rmwaistop1-rmwaistop4, covname(rmwaist) at(68.58 69.215 69.342 69.85 70.485 71.12 71.755 72.39 73.025 73.66 74.295 74.676 74.93 75.565 75.6412 76.2 76.835 77.47 78.105 78.232 78.74 79.248 79.375 80.01 80.645 81.28 81.788 81.915 82.55 82.804 83.185 83.82 84.455 85.09 85.725 86.36 86.741 86.995 87.63 87.884 88.265 88.9 88.9508 89.408 89.535 89.789 90.17 90.805 91.44 91.567 91.7702 91.948 92.075 92.71 92.964 93.218 93.345 93.472 93.98 94.0054 94.0308 94.107 94.234 94.615 95.25 95.885 96.52 96.647 97.155 97.2312 97.3836 97.79 97.917 98.425 99.06 99.187 99.441 99.568 99.695 100.33 100.965 101.6 101.727 101.854 102.108 102.235 102.87 102.997 103.378 103.505 104.14 104.267 104.775 105.029 105.156 105.41 106.045 106.426 106.68 106.807 106.934 107.188 107.315 107.696 108.585 109.22 109.347 109.474 109.855 110.49 110.998 111.125 111.252 111.76 111.887 112.395 113.03 113.665 114.3 114.554 114.935 115.062 115.57 116.205 116.84 117.348 117.475 117.602 118.11 118.745 119.38 119.507 119.634 119.888 120.015 120.65 121.285 121.92 122.301 122.555 123.19 123.825 124.46 124.587 124.714 125.095 125.73 126.365 127 127.127 127.635 128.016 128.27 128.905 129.54 129.794 130.175 130.81 131.445 132.08 132.715 133.35 133.985 134.62 135.255 135.89 136.525 137.16 137.541 137.795 138.43 139.065 139.7) reference(95.25) eform generate(pa or lb ub)*

*twoway rarea lb ub pa, bcolor(gs14) ///*

*|| line or pa, clcolor(red) clwidth(medthick) ///*

*, ylabel(0(5)10, angle(0) nogrid) xlabel(70 (10) 140) ///*

*xtitle(Waist Circumference (cm)) ytitle(OR (95%CI)) title(Female) ///*

*name(female,replace) legend(order(2 "Expected OR" 1 "95% CI") rows(1))*

*graph combine female male,title("Association between UI and WC by Sex" "(in HRS study)")*

*graph export "hrs-rcs-waist.png",replace*
