# Supplementary material for: Association between obesity and urinary incontinence in older adults from multiple nationwide longitudinal cohorts
Source: Commun Med (Lond). 2023 Oct 11;3:142. doi: 10.1038/s43856-023-00367-w (PMC10567749; doi:10.1038/s43856-023-00367-w)
Supplement: Supplementary file 4 — Supplementary Information [file 43856_2023_367_MOESM4_ESM.pdf]

Supplementary Figure 1. Association between UI and BMI by age group by male and female in HRS study, 2010-2018

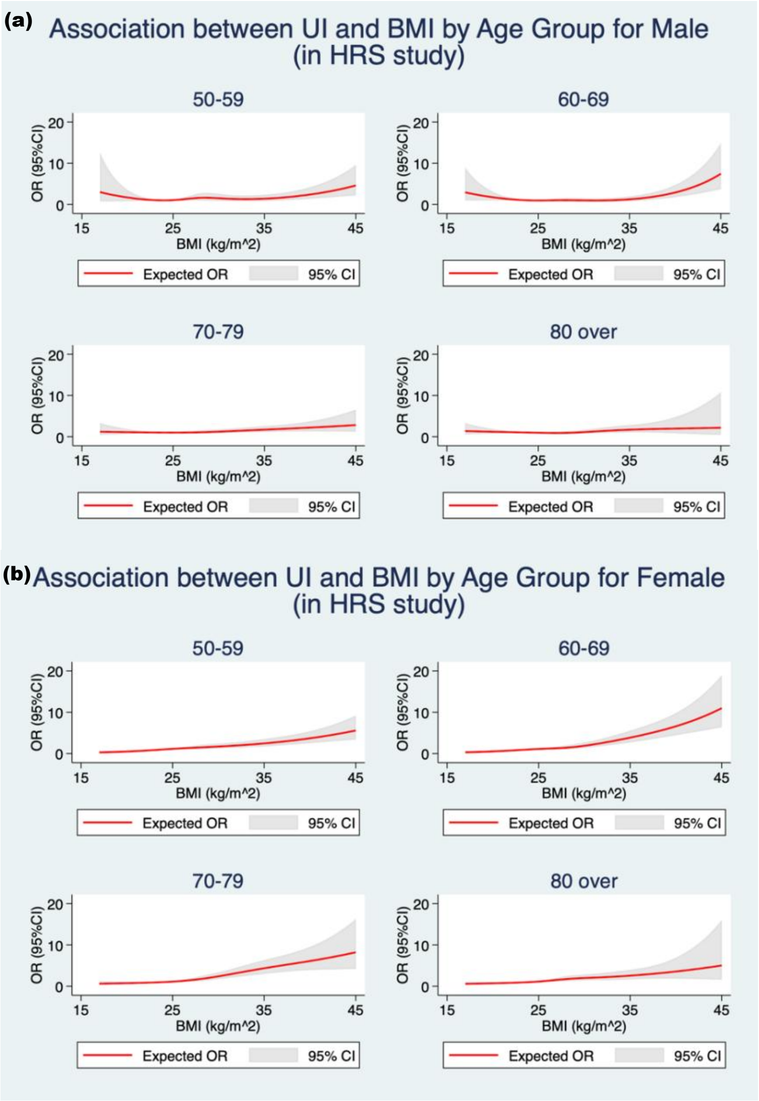

(a) Association between UI and BMI by age group (50-59 years group/60-69 years group/70-79 years group/80 over years group) by male in HRS study. (b) Association between UI and BMI by age group (50-59 years group/60-69 years group/70-79 years group/80 over years group) by female in HRS study. Red solid lines, expected odds ratio; Grey shades, 95% confidence interval. HRS, the Health and Retirement Study; BMI, body mass index; UI, urinary incontinence; OR, odds ratio; 95%CI, 95% confidence interval.

Supplementary Figure 2. Association between UI and WC by age group by male and female in HRS study, 2010-2018

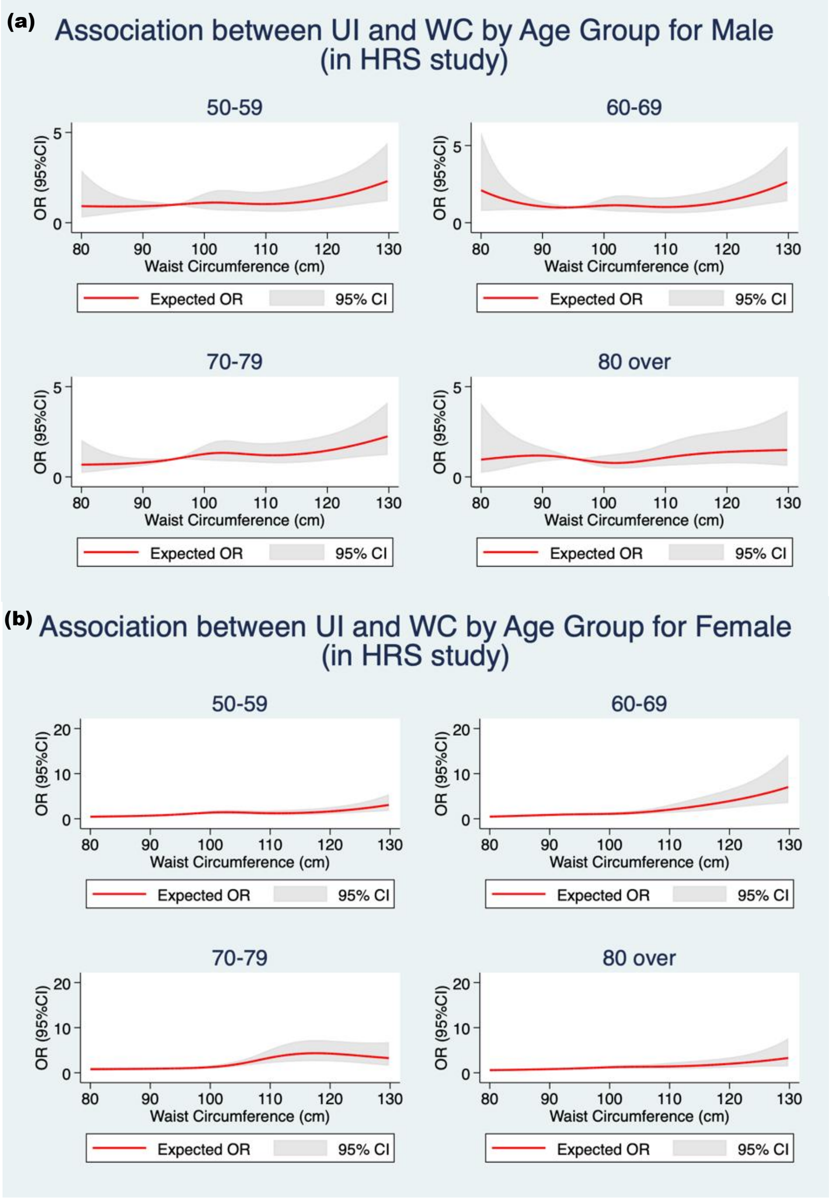

(a) Association between UI and WC by age group (50-59 years group/60-69 years group/70-79 years group/80 over years group) by male in HRS study. (b) Association between UI and WC by age group (50-59 years group/60-69 years group/70-79 years group/80 over years group) by female in HRS study. Red solid lines, expected odds ratio; Grey shades, 95% confidence interval. HRS, the Health and Retirement Study; WC, waist circumferences; UI, urinary incontinence; OR, odds ratio; 95%CI, 95% confidence interval.

Supplementary Figure 3. Association between UI and BMI by age group by male and female in SHARE study, 2004-2010 (except 2018)

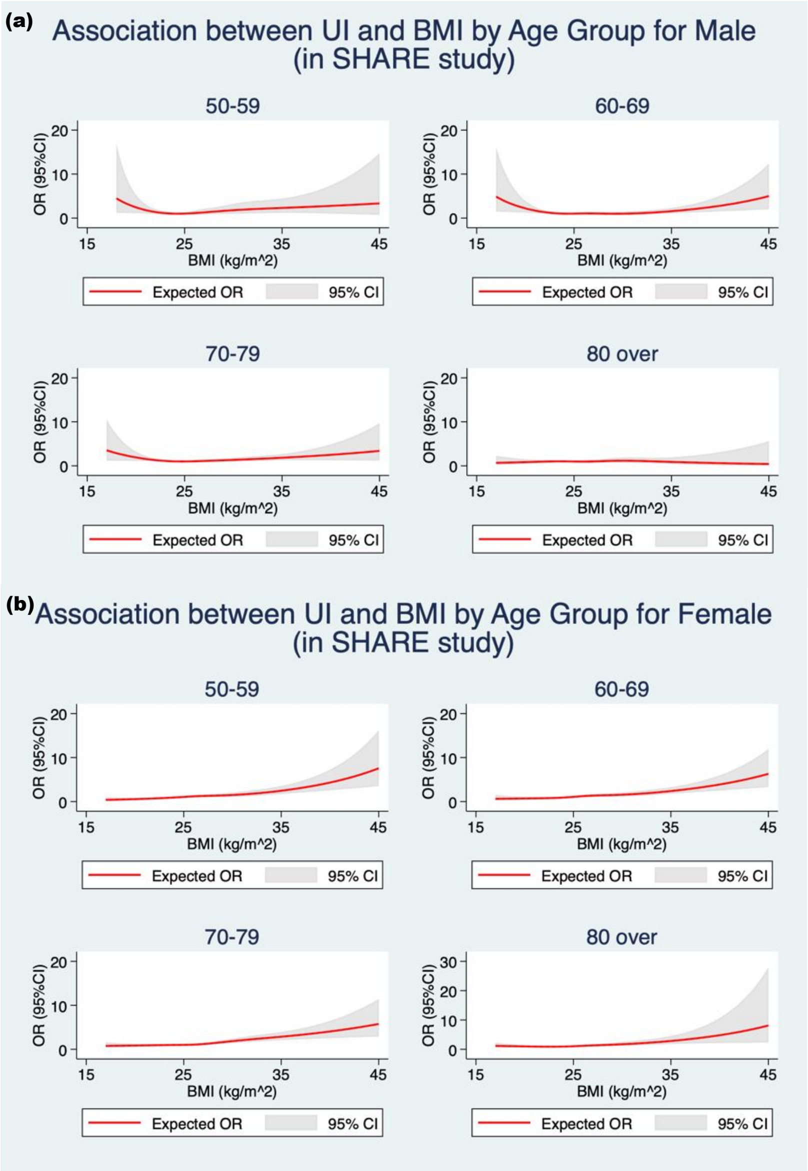

(a) Association between UI and BMI by age group (50-59 years group/60-69 years group/70-79 years group/80 over years group) by male in SHARE study. (b) Association between UI and BMI by age group (50-59 years group/60-69 years group/70-79 years group/80 over years group) by female in SHARE study. Red solid lines, expected odds ratio; Grey shades, 95% confidence interval. SHARE, Survey of Health, Ageing and Retirement in Europe; BMI, body mass index; UI, urinary incontinence; OR, odds ratio; 95%CI, 95% confidence interval.

Supplementary Table 1. Association between BMI and waist circumferences, and prevalence of urinary incontinence by gender and race, HRS 2010-2018

Variables	Female						Male					
	White/Caucasion		Black/African American		Other		White/Caucasion		Black/African American		Other	
	OR (95% CI)	p value	OR (95% CI)	p value	OR (95% CI)	p value	OR (95% CI)	p value	OR (95% CI)	p value	OR (95% CI)	p value
BMI (6 quantiles)												
Quantile 1	0.599(0.499-0.719)	<0.001	0.581(0.314-1.075)	0.084	0.576(0.301-1.103)	0.096	1.291(0.980-1.701)	0.069	1.190(0.671-2.111)	0.551	1.578(0.671-3.707)	0.295
Quantile 2	Reference	-	Reference	-	Reference	-	Reference	-	Reference	-	Reference	-
Quantile 3	1.068(0.896-1.272)	0.462	0.775(0.458-1.311)	0.341	1.142(0.636-2.053)	0.656	1.168(0.927-1.471)	0.187	1.089(0.663-1.788)	0.738	0.838(0.409-1.719)	0.630
Quantile 4	1.478(1.217-1.795)	<0.001	0.931(0.543-1.597)	0.795	1.555(0.847-2.856)	0.154	1.071(0.831-1.379)	0.598	0.909(0.530-1.560)	0.730	1.267(0.597-2.692)	0.537
Quantile 5	2.079(1.679-2.573)	<0.001	1.211(0.706-2.076)	0.487	1.803(0.948-3.430)	0.072	1.322(1.004-1.741)	0.047	1.118(0.639-1.956)	0.695	1.144(0.521-2.511)	0.738
Quantile 6	3.423(2.689-4.356)	<0.001	1.798(1.047-3.087)	0.033	2.643(1.358-5.144)	0.004	2.039(1.491-2.790)	<0.001	1.400(0.764-2.566)	0.277	1.870(0.823-4.247)	0.135
WC (6 quantiles)												
Quantile 1	0.606(0.472-0.778)	<0.001	1.665(0.784-3.538)	0.185	0.949(0.429-2.103)	0.898	1.067(0.602-1.891)	0.824	0.914(0.339-2.468)	0.859	0.990(0.366-2.683)	0.985
Quantile 2	Reference	-	Reference	-	Reference	-	Reference	-	Reference	-	Reference	-
Quantile 3	1.217(0.940-1.574)	0.136	1.454(0.695-3.041)	0.320	2.057(0.947-4.472)	0.069	1.302(0.905-1.875)	0.156	0.703(0.312-1.583)	0.395	1.026(0.492-2.140)	0.945
Quantile 4	1.457(1.084-1.960)	0.013	1.920(0.878-4.195)	0.102	5.062(2.094-12.239)	<0.001	1.101(0.743-1.633)	0.631	0.410(0.159-1.061)	0.066	0.767(0.326-1.801)	0.542
Quantile 5	2.361(1.740-3.203)	<0.001	1.708(0.792-3.684)	0.172	2.235(0.917-5.446)	0.077	1.265(0.862-1.856)	0.229	0.735(0.313-1.727)	0.480	0.850(0..387-1.867)	0.687
Quantile 6	3.267(2.293-4.654)	<0.001	3.003(1.374-6.564)	0.006	3.809(1.415-10.252)	0.008	1.955(1.306-2.927)	0.001	0.770(0.320-1.858)	0.561	1.045(0.458-2.382)	0.917

HRS, the Health and Retirement Study; BMI, body mass index; WC, waist circumference; OR, odds ratio; 95% CI, 95% confidence interval.
