# Supplementary material for: Association between obesity and urinary incontinence in older adults from multiple nationwide longitudinal cohorts
Source: Commun Med (Lond). 2023 Oct 11;3:142. doi: 10.1038/s43856-023-00367-w (PMC10567749; doi:10.1038/s43856-023-00367-w)
Supplement: Supplementary file 5 — Description of Additional Supplementary Files [file 43856_2023_367_MOESM5_ESM.pdf]

Description of Additional Supplementary Files

File Name: Supplementary Data 1

Description: BMI, waist circumferences, and covariates of observations by sex in analyses

File Name: Supplementary Data 2

Description: Association between BMI and waist circumferences, and prevalence of urinary incontinence

File Name: Supplementary Data 3

Description: Key STATA codes for the manuscript “Association between obesity and urinary incontinence in older adults from multiple nationwide longitudinal cohorts
