# Supplementary material for: Association between obesity and urinary incontinence in older adults from multiple nationwide longitudinal cohorts
Source: Commun Med (Lond). 2023 Oct 11;3:142. doi: 10.1038/s43856-023-00367-w (PMC10567749; doi:10.1038/s43856-023-00367-w)
Supplement: Supplementary file 7 — Reporting Summary [file 43856_2023_367_MOESM7_ESM.pdf]

Corresponding author(s): Yao Yao

Last updated by author(s): Jul 27, 2023

Reporting Summary

Nature Portfolio wishes to improve the reproducibility of the work that we publish. This form provides structure for consistency and transparency in reporting. For further information on Nature Portfolio policies, see our Editorial Policies and the Editorial Policy Checklist.

Statistics

For all statistical analyses, confirm that the following items are present in the figure legend, table legend, main text, or Methods section.

n/a	Confirmed	
☐	☒	The exact sample size (*n*) for each experimental group/condition, given as a discrete number and unit of measurement
☐	☒	A statement on whether measurements were taken from distinct samples or whether the same sample was measured repeatedly
☐	☒	The statistical test(s) used AND whether they are one- or two-sided<br>*Only common tests should be described solely by name; describe more complex techniques in the Methods section.*
☐	☒	A description of all covariates tested
☐	☒	A description of any assumptions or corrections, such as tests of normality and adjustment for multiple comparisons
☐	☒	A full description of the statistical parameters including central tendency (e.g. means) or other basic estimates (e.g. regression coefficient) AND variation (e.g. standard deviation) or associated estimates of uncertainty (e.g. confidence intervals)
☐	☒	For null hypothesis testing, the test statistic (e.g. *F*, *t*, *r*) with confidence intervals, effect sizes, degrees of freedom and *P* value noted<br>*Give P values as exact values whenever suitable.*
☒	☐	For Bayesian analysis, information on the choice of priors and Markov chain Monte Carlo settings
☒	☐	For hierarchical and complex designs, identification of the appropriate level for tests and full reporting of outcomes
☒	☐	Estimates of effect sizes (e.g. Cohen's *d*, Pearson's *r*), indicating how they were calculated

Our web collection on statistics for biologists contains articles on many of the points above.

Software and code

Policy information about availability of computer code

Data collection	Data were accessed from three international cohorts of aging: Health and Retirement Study (HRS), English Longitudinal Study of Ageing (ELSA), Survey of Health, Ageing and Retirement in Europe (SHARE), which were used to provide the representative sample and comparable measures on BMI, WC, UI, and other covariates, covering 21 countries including both developed and developing ones on two continents. ELSA received ethical approval by the London Multicentre Research Ethics Committee (MREC/01/2/91). The University of Mannheim's internal review board (IRB) reviewed and approved SHARE for wave 1 to 4. From wave 4 onwards, the ethics reviews were done by Ethics Council of the Max Planck Society. The detail of the ethics approvals is available from SHARE project's website (http://www.share-project.org/fileadmin/pdf_documentation/SHARE_ethics_approvals.pdf). The HRS was approved by the University of Michigan Institutional Review Board (HUM00061128). The secondary data analysis of the above cohorts, such as was done in the current study, underwent no further ethical approval. We have obtained permission to access and utilize three longitudinal datasets by registration and then application, and have published a series of articles already. As with previous articles, no individual reference license numbers were provided. Meanwhile, a reference number for conducting the secondary analysis is not required by Peking University.
Data analysis	All analyses and plots were performed using STATA 17.0. We do not make our codes publicly available at this point due to our other ongoing series of research related to urinary incontinence. However, our computer codes are accessible to researchers upon reasonable request to the corresponding author. Our computer code are accessible to researchers upon request to the corresponding author.

For manuscripts utilizing custom algorithms or software that are central to the research but not yet described in published literature, software must be made available to editors and reviewers. We strongly encourage code deposition in a community repository (e.g. GitHub). See the Nature Portfolio guidelines for submitting code & software for further information.

Data

Policy information about availability of data

All manuscripts must include a data availability statement. This statement should provide the following information, where applicable:

- Accession codes, unique identifiers, or web links for publicly available datasets
- A description of any restrictions on data availability
- For clinical datasets or third party data, please ensure that the statement adheres to our policy

The original survey datasets used and analyzed are publicly available, which are also provided with this paper. These datasets that support the findings of this study are available from the GATEWAY TO GLOBAL AGING DATA (https://g2aging.org/) upon registration.

Human research participants

Policy information about studies involving human research participants and Sex and Gender in Research.

Reporting on sex and gender	Our findings do not apply to only one sex or gender. Sex and gender were considered in our study design, and sex of participants was determined based on self-report through questionnaire. Of enrolled participants' observations, the females were responsible for 55.7% in the HRS study, 54.3% in the ELSA study, and 55.1% in the SHARE study, respectively. In this study, data was reported disaggregated for sex where this information has been collected and consent has been obtained for reporting and sharing individual-level data. Sex based analyses were conducted and results were reported.
Population characteristics	See above
Recruitment	Data were accessed from three international cohorts of aging: Health and Retirement Study (HRS), English Longitudinal Study of Ageing (ELSA), Survey of Health, Ageing and Retirement in Europe (SHARE). Given the availability of UI measurements and similar time ranges, we used data from the following time period in this analysis: 2010–2018 for HRS, 2010–2018 for ELSA, and 2004–2010 for SHARE. Participants who were younger than 50 years old were excluded.
Ethics oversight	All data were collected from administrative databases, and therefore no additional ethical approval was needed for this study.

Note that full information on the approval of the study protocol must also be provided in the manuscript.

Field-specific reporting

Please select the one below that is the best fit for your research. If you are not sure, read the appropriate sections before making your selection.

☐ Life sciences ☒ Behavioural & social sciences ☐ Ecological, evolutionary & environmental sciences

For a reference copy of the document with all sections, see nature.com/documents/nr-reporting-summary-flat.pdf

Behavioural & social sciences study design

All studies must disclose on these points even when the disclosure is negative.

Study description	Findings from three prospective longitudinal cohort studies towards BMI, waist circumferences and urinary incontinence in older women compared with older men
Research sample	Three international cohorts of aging: Health and Retirement Study (HRS), English Longitudinal Study of Ageing (ELSA), Survey of Health, Ageing and Retirement in Europe (SHARE), which were used to provide the representative sample and comparable measures on BMI, WC, UI, and other covariates, covering 21 countries including both developed and developing ones on two continents. Original survey datasets from HRS, ELSA, and SHARE are freely available to all bona fide researchers upon requesting. The data that support the findings of this study are available from the GATEWAY TO GLOBAL AGING DATA (https://g2aging.org/). We used data from the following time period in this analysis: 2010–2018 for HRS, 2010–2018 for ELSA, and 2004–2010 for SHARE.
Sampling strategy	Data were drawn from three international cohorts of aging: Health and Retirement Study (HRS), English Longitudinal Study of Ageing (ELSA), Survey of Health, Ageing and Retirement in Europe (SHARE).
Data collection	Data on urinary incontinence, BMI (kg/m2), waist circumference (cm), and covariates (age, race, residence area, marital status, number of children, smoking, drinking, hypertension, diabetes, cancer, stroke as well as educational attainments, functional ability, and cognitive impairment) were accessed from three international cohorts of aging for those who are over 50 years old: Health and Retirement Study (HRS), English Longitudinal Study of Ageing (ELSA), Survey of Health, Ageing and Retirement in Europe (SHARE), which were used to provide the representative sample and comparable measures on BMI, WC, UI, and other covariates, covering 21 countries including both developed and developing ones on two continents. Given the availability of UI measurements and similar

	time ranges, we used data from the following time period in this analysis: 2010–2018 for HRS, 2010–2018 for ELSA, and 2004–2010 for SHARE.
Timing	Given the availability of UI measurements and similar time ranges, we used data from the following time period in this analysis: 2010–2018 for HRS, 2010–2018 for ELSA, and 2004–2010 for SHARE.
Data exclusions	Participants who were younger than 50 years old were excluded. The rationale for this exclusion is that this study focuses on older population. And the exclusion criteria were pre-established.
Non-participation	Following the exclusion criteria, 121,450 SHARE participants with 360,800 observations, 19,791 ELSA participants with 98,158 observations, and 42,132 HRS participants with 207,805 observations were analyzed. The rationale for this exclusion is that this study focuses on older population.
Randomization	No experiments were used in this study and, therefore, no randomization was used.

Reporting for specific materials, systems and methods

We require information from authors about some types of materials, experimental systems and methods used in many studies. Here, indicate whether each material, system or method listed is relevant to your study. If you are not sure if a list item applies to your research, read the appropriate section before selecting a response.

Materials & experimental systems

n/a	Involved in the study
☒	☐ Antibodies
☒	☐ Eukaryotic cell lines
☒	☐ Palaeontology and archaeology
☒	☐ Animals and other organisms
☒	☐ Clinical data
☒	☐ Dual use research of concern

Methods

n/a	Involved in the study
☒	☐ ChIP-seq
☒	☐ Flow cytometry
☒	☐ MRI-based neuroimaging
